# The Preparation of Novel P(OEGMA-co-MEO_2_MA) Microgels-Based Thermosensitive Hydrogel and Its Application in Three-Dimensional Cell Scaffold

**DOI:** 10.3390/gels8050313

**Published:** 2022-05-19

**Authors:** Yang Liu, Yu-Ning Luo, Pei Zhang, Wen-Fei Yang, Cai-Yao Zhang, Yu-Li Yin

**Affiliations:** Hunan Provincial Key Laboratory of Tumor Microenvironment Responsive Drug Research, Hunan Province Cooperative Innovation Center for Molecular Target New Drug Study, School of Pharmacy, Hengyang Medical School, University of South China, Hengyang 421001, China; luo1748058392@163.com (Y.-N.L.); zp1832711702@163.com (P.Z.); yang18138800314@163.com (W.-F.Y.); caiyao.zhang@nuclover.com (C.-Y.Z.); yinyulimy@163.com (Y.-L.Y.)

**Keywords:** thermosensitive, hydrogel, P(OEGMA-co-MEO_2_MA), microgel, scaffold, 3D culture

## Abstract

Thermosensitive hydrogel scaffolds have attracted particular attention in three-dimensional (3D) cell culture. It is very necessary to develop a type of thermosensitive hydrogel material with low shrinkage, and excellent biocompatibility and biodegradability. Here, five types of thermosensitive microgels with different volume phase transition temperature (VPTT) or particle sizes were first synthesized using 2-methyl-2-propenoic acid-2-(2-methoxyethoxy) ethyl ester (MEO_2_MA) and oligoethylene glycol methyl ether methacrylate (OEGMA) as thermosensitive monomers by free radical polymerization. Their VPTT and particle sizes were investigated by a nanometer particle size meter and an ultraviolet spectrophotometer. The feasibility of using these P(OEGMA-co-MEO_2_MA) microgels to construct thermosensitive hydrogel by means of the thermal induction method is discussed for the first time. The prepared thermosensitive hydrogel with the optimum performance was screened for in situ embedding and three-dimensional (3D) culture of MCF-7 breast cancer cells. The experimental results of AO/EB and MTT methods indicate that the pioneering scaffold material has prominent biocompatibility, and cells grow rapidly in the 3D scaffold and maintain high proliferative capacity. At the same time, there is also a tendency to aggregate to form multicellular spheres. Therefore, this original P(OEGMA-co-MEO_2_MA) thermosensitive hydrogel can serve as a highly biocompatible and easily functionalized 3D cell culture platform with great potential in the biomedical area.

## 1. Introduction

In recent years, the use of tissue engineering technology for in vitro three-dimensional (3D) cell culture to construct 3D cell models for tumor research, drug screening, and tissue repair has aroused great interest in biomedical researchers and clinical workers [1,2,3]. Among them, scaffold materials play a key role. As the carrier of seed cells and bioactive growth factors, scaffold materials can provide a favorable microenvironment for cell growth, reproduction, metabolism, and other physiological activities, and induce cells to form a 3D model [4,5,6,7]. Therefore, an excellent scaffold material should have a 3D porous network structure to facilitate cell insertion and transport of nutrients and metabolic waste. At the same time, it should have ideal biocompatibility, degradability and mechanical strength matching the properties of the extracellular matrix.

Hydrogel is a kind of soft material with a 3D network structure, formed through various physical or chemical crosslinking methods [8,9,10,11]. Hydrogels have extremely similar physical properties to extracellular matrices, which have been widely used as scaffold materials for 3D cell culture [12,13,14]. Among them, thermosensitive polymers-based hydrogel scaffolds have attracted particular attention [15,16,17]. Thermosensitive polymers generally appear liquid at room temperature, and can be evenly mixed with cells, drugs, growth factors, etc. When the temperature rises to its low critical dissolution temperature (LCST), or volume phase transition temperature (VPTT), and appropriate ions are present, the thermosensitive polymer undergoes in situ gelation to form injectable 3D hydrogels. Thus, it can nondestructively embed cells in situ and promote further cell growth. By cooling it to liquefy it, cells, or complex cell aggregates, can also be easily released. 

Poly(N-isopropylacrylamide) (PNIPAM) is the most studied thermosensitive material with LCST or VPTT of 32 °C, and is often used as a scaffold material [18,19,20]. For instance, Ekerdt and colleagues successfully constructed a type of thermosensitive hyaluronic acid (HA)-PNIPAM brush polymer through thiol-ene click chemistry, which could mix with human embryonic stem cells (hESCs), or human induced pluripotent stem cells (hiPSCs), at 4 °C, and gel rapidly to form a hydrogel in situ at 37 °C [21]. After five days of culture, cells were able to grow rapidly and aggregate to form regular multicellular spheroids, while maintaining cell pluripotency after multiple passages. Moreover, this type of hydrogel was able to release the cells after cooling, liquefaction and centrifugation [22]. Liang et al. also synthesized a type of dendritic thermosensitive polymer from PNIPAM, dimeric glycerol, and polyethylene glycol (PEG). This polymer could also be used as a scaffolding material for the culture of hiPSCs, which were similarly able to release cells after simple cooling, liquefaction and centrifugation [23].

To further adjust and improve the properties of formed thermosensitive hydrogel, spherical thermosensitive polymers, namely microgels, were also used for the fabrication of hydrogel scaffolds. Microgels are spherical hydrogel nanoparticles with particle sizes from 10 nm to a few microns, which have the advantages of simple preparation, fast response and easy modification and are of considerable interest in the biomedical field [24,25,26,27]. Gan et al. successfully constructed this type of thermosensitive hydrogel scaffold using PNIPAM-based thermosensitive microgels for the first time. The hydrogel thus formed has an interconnected porous structure, which is highly conducive to the transport of oxygen, nutrients and cellular metabolites. Human embryonic kidney (HEK) 293T cells could be encapsulated in this hydrogel and grew well [28]. We also used PNIPAM-based microgels as scaffold materials to encapsulate aggregates of Human hepatocellular carcinomas HepG2 cells, in which multicellular spheroids of HepG2 cells could be quickly obtained [29]. In addition, Shen et al. also used the more hydrophilic NIPAM and acrylamide (AAm) copolymer microgels as scaffold materials for the culture of mouse melanoma cells [30]. It was found that the cells gradually grew in a single dispersed state. The larger the particle size of the microgels used, the better the activity of the cells. The Dai group also used PNIPAM-based microgels with a small negative charge and a small positive charge as cell scaffolds to study the culture and behavior of mouse embryonic mesenchymal stem cells (MSCS), respectively [31,32]. 

Despite the numerous advantages of PNIPAM thermosensitive polymers, the thermosensitive hydrogels formed from them suffer from a certain degree of shrinkage over time, which severely limits cell growth and activity. Although it is possible to reduce the shrinkage of the hydrogels by introducing acrylic acid (AA) with negative charge [33], blending PEG [29,34] and adjusting the particle size of the microgels [35], the effect is still not satisfactory. In addition, PNIPAM polymers are toxic to some extent and difficult to degrade. Therefore, it is an urgent problem to find a type of thermosensitive gel material with low shrinkage, and excellent biocompatibility and biodegradability. In recent years, novel thermosensitive polymers based on oligo(ethylene glycol) methacrylate (OEGMA) and 2-(2-methoxyethoxy) ethyl acrylate (MEO_2_MA) have aroused great interest from researchers because of their excellent biocompatibility and biodegradability [36,37]. OEGMA and MEO_2_MA are two types of PEG-based macromolecular monomers with similar structures, in which the LCST of POEGMA homopolymer is about 90 °C and often acts as a hydrophilic chain segment, while PMEO_2_MA homopolymer has an LCST of about 19 °C and often acts as a hydrophobic chain segment. The copolymers formed from them also have outstanding thermosensitive behavior. The LCST or VPTT could vary from 19 to 90 °C by simply adjusting the relative proportions of OEGMA and MEO_2_MA in the polymerization process. Several studies have reported using these novel thermosensitive polymers for the culture of various types of cells. For instance, Anderson et al. prepared a brush-like P(OEGMA-co-MEO_2_MA) thermosensitive polymer by adjusting the ratio of OEGMA and MEO_2_MA. It was used as a matrix material to study the effect on adhesion and morphology of L-929 mouse fibroblasts [38,39]. It was found that the combination of thermosensitive polymers with a smaller relative proportion of hydrophilic OEGMA was more favorable for cell growth and adhesion. In addition, Bakaic et al. [40] constructed an injectable hydrogel scaffold with temperature and pH dual responsiveness using chemical crosslinking between neutral or charged aldehyde functionalized POEGMA copolymers and hydrazide functionalized POEGMA copolymer precursors. They found that the charged hydrogel was more supportive of the two-dimensional (2D) cell adhesive of 3T3 mouse fibroblasts and the 3D stabilization and proliferative of ARPE-19 human retinal epithelial cells.

In this manuscript, we discuss the feasibility of using P(OEGMA-co-MEO_2_MA) spherical microgels to construct thermosensitive hydrogel 3D cell scaffolds for the first time. We first used OEGMA and MEO_2_MA to prepare a series of P(OEGMA-co-MEO_2_MA) microgels with different VPTT and sizes through free radical polymerization. Then, novel P(OEGMA-co-MEO_2_MA) microgels-based thermosensitive hydrogels were prepared by the thermal induction method under conditions of heating and the presence of salts. MCF-7 human breast cancer cells were used as the model cells for in-situ embedding and 3D co-culture in this novel thermosensitive hydrogel. To the best of our knowledge, there are no reports related to P(OEGMA-co-MEO_2_MA)-based microgels as scaffolds for 3D cell culture. Therefore, our study may provide new ideas for the development of thermosensitive hydrogel scaffolds.

## 2. Results and Discussion

### 2.1. The Synthesis of P(OEGMA-co-MEO_2_MA) Thermosensitive Microgels

In this work, to investigate the influence of the properties of microgels on subsequent hydrogel formation, five types of P(OEGMA-co-MEO_2_MA) microgel samples, with different thermosensitivities or sizes, were synthesized by a free radical polymerization reaction [26].

**Figure 1:**
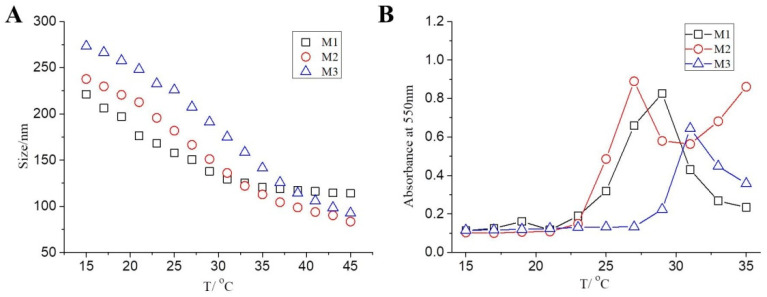
Hydrodynamic radius (**A**) and absorbance (**B**) of P(OEGMA-co-MEO_2_MA) microgel samples M1-M3 as a function of temperature.

As shown in Table 1, we first synthesized M1-M3 samples through changing the mass ratio of thermosensitive monomers OEGMA and MEO_2_MA. We measured the particle sizes of microgel aqueous solutions at different temperatures using a nanometer particle size meter. It can be seen from Figure 1A that the particle sizes of all the three P(OEGMA-co-MEO_2_MA) microgels decreased gradually with increase in temperature, which indicated that all of them had great thermosensitivity, similar to linear and branched P(OEGMA-co-MEO_2_MA) polymers. At lower temperatures, due to the strong hydrogen bonding of oxygen atoms in the microgel, the microgel particle has strong hydrophilicity and high water content, so the particle size is larger, which is manifested as volume swelling. When the temperature gradually increases, the hydrogen bond is gradually destroyed, and the microgel gradually shows strong hydrophobic effect derived from alkyl groups, so the internal water content decreases, the volume shrinks, and the particle size decreases. The temperature at which the slope of the curve changes the most is defined as the VPTT. As shown in the figure, the VPTT of M1, M2, and M3 were about 21 °C, 2 °C, and 2 °C, respectively, which means that with the increase of the relative proportion of OEGMA in the monomers, the final microgel has a higher VPTT. This is because, compared with MEO_2_MA, OEGMA has more ether oxygen bonds in the molecular structure, which can form more hydrogen bonds with water molecules; so, the more OEGMA content, the stronger the hydrophilicity of the formed P(OEGMA-co-MEO_2_MA) microgel. In addition, we found that the sizes of M3 were larger than others at most temperatures, which may also be due to the higher content of OEGMA increasing the hydrophilicity and swelling of this microgel. This is in agreement with what was reported in the literature [41].

In order to further study the thermosensitivity of the above prepared P(OEGMA-co-MEO_2_MA) microgels, we used phosphate buffer solution (PBS) to configure a certain concentrated dilute solution of microgel samples, and measured the change of absorbance at different temperatures by using an UV-Vis spectrophotometer. As shown in Figure 1B, when the temperature was lower, the absorbance of all the three microgel solutions was basically the same, but when the temperature exceeded a certain higher critical value, it mutated suddenly. The reason for this phenomenon is that with increase of temperature, hydrophilic microgels gradually become hydrophobic. When a critical temperature is reached, microgels gather together to form small aggregates, resulting in increased light refraction, thus decreasing light transmittance and increasing absorbance. Actually, the critical temperature is the VPTT. Similarly, we found that the VPTT of three samples was 21 °C, 23 °C and 27 °C, respectively. With increase of the relative proportion of OEGMA, the VPTTs of the samples M1, M2, M3 increased gradually, which was consistent with previous results.

**Figure 2:**
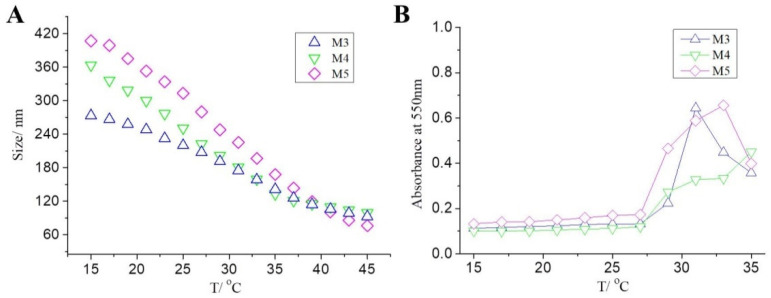
Hydrodynamic radius (**A**) and absorbance (**B**) of P(OEGMA-co-MEO_2_MA) microgel samples M3-M5 as a function of temperature.

In addition, the effect of the dosage of surfactant sodium dodecyl sulfate (SDS) on P(OEGMA-co-MEO_2_MA) microgel properties was also investigated. We synthesized microgel samples M4 and M5, and compared them with sample M3. The SDS dosages in their synthesis processes were reduced (Table 1). We first also measured the particle sizes of microgel solutions at different temperatures. As shown in Figure 2A, the particle sizes of M4 and M5 also decreased gradually with increase of temperature, which indicated the presence of excellent thermosensitivity. From M3, M4 to M5, with reduction of the SDS dosages, the sizes of the obtained microgels increased at measured temperatures, especially at lower temperatures. For instance, at 15 °C, the sizes of M3, M4 and M5 were 273.3 nm, 363.5 nm, and 406.7 nm, respectively. Moreover, we found that all these three types of microgel samples had the same VPTT, around 27 °C. The results of absorbance measurements at different temperatures, shown in Figure 2B, also indicates that the VPTT remains constant regardless of the SDS dosage. These phenomena indicated that with the decrease of SDS dosage, the relative particle size of the microgel generally increased, but the VPTT remained basically unchanged. This is because SDS in the reaction process does not directly participate in the polymerization reaction, but plays the role of stabilizing the parent particles of microgel by forming micelles. So, the SDS dosage will not affect the thermosensitivities and VPTTs of obtained microgels. The lower the SDS content, the larger the size of the micelle formed from the SDS, so the size of the final P(OEGMA-co-MEO_2_MA) microgel particle is larger.

In summary, three types of thermosensitive P(OEGMA-co-MEO_2_MA) microgels, with different VPTTs, were synthesized by free radical emulsion polymerization through change in the mass ratio of OEGMA and MEO_2_MA. With increase of the content of OEGMA, the VPTT of the obtained microgels also increased. In addition, two types of thermosensitive P(OEGMA-co-MEO_2_MA) microgels, with different sizes and the same VPTT, were also synthesized by polymerization through change of the SDS dosage. With the decrease of the dosages of SDS, the sizes of the obtained microgels increased.

### 2.2. The Preparation of P(OEGMA-co-MEO_2_MA) Thermosensitive Microgels-Based Hydrogel

Next, we studied the feasibility of using P(OEGMA-co-MEO_2_MA) microgels to construct thermosensitive hydrogels through the thermal induction method. Referring to our previously reported method [29], we configured the PBS solutions of microgel samples with a concentration of 3.0 wt%, and placed them at 37 °C, which was higher than their VPTT. Then, we observed the formation of hydrogels by the inverted method and photographed the changes of these hydrogels.

**Figure 3:**
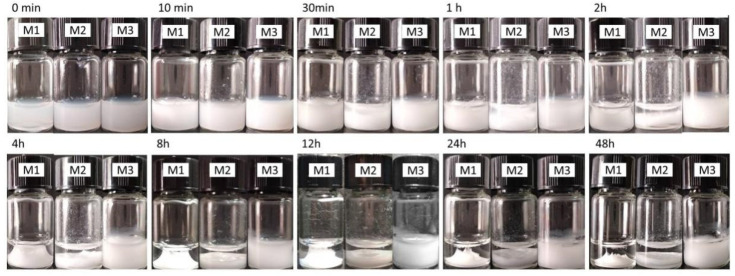
Photographs of the P(OEGMA-co-MEO_2_MA) hydrogels formed from M1–M3 microgels at different times.

As shown in Figure 3, after maintaining for 10 min at 37 °C, all three types of P(OEGMA-co-MEO_2_MA) microgels could gel and form white hydrogels. This result was consistent with that of the traditional PNIPAM thermosensitive polymer. When the temperature was higher than their VPTT, the microgels, in their hydrophobic contraction state, would aggregate under the synergistic influence of hydrophobicity and the shielding effect of ions from PBS, thus forming hydrogel macroscopically. The gelation times were about 5 min to 10 min. Meanwhile, we found that the hydrogels formed from M1 and M2 showed obvious shrinkage after 2 h, and the shrinkage degree increased with time. After 48 h, the volume of the hydrogels formed from M1 and M2 was less than 1/2 of the initial value. This phenomenon of volume shrinkage has been reported several times regarding traditional PNIPAMs and other thermosensitive hydrogels, because of the dehydration of thermosensitive materials at higher temperature [19,20]. As an ideal scaffold material, the volume contraction should be avoided as far as possible in order to maintain sufficient cell growth space and ensure the circulation of nutrients and metabolic waste [4,6].

**Figure 4:**
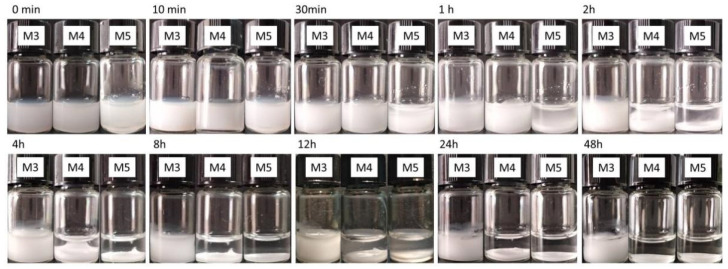
Photographs of the P(OEGMA-co-MEO_2_MA) hydrogels formed from M3–M5 microgels at different times.

Encouragingly, we found that the volume change of the hydrogel formed by the prepared sample M3 with a higher VPTT was less than 5% and no significant shrinkage was observed. So, we also observed and recorded the formation of hydrogels from M4 and M5 microgels with the same VPTT and larger sizes. It can be seen from Figure 4 that the hydrogels formed from M4 and M5 also showed obvious shrinkage after 2 h, and the shrinkage degree increased with time, which was hardly surprising.

**Figure 5:**
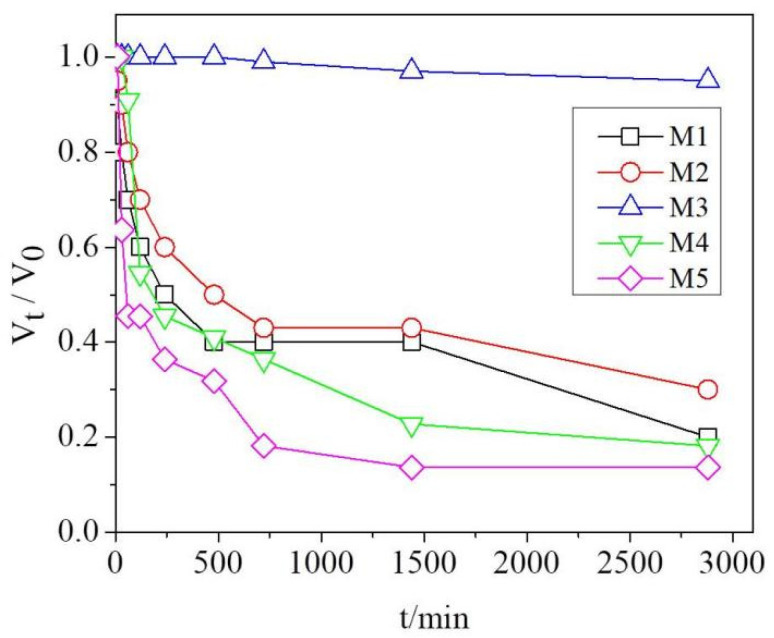
The change of V_t_/V_0_ (shrinkage degree) of P(OEGMA-co-MEO_2_MA) hydrogels formed from microgels as a function of time.

We further investigated the quantitative relation between the volume shrinkage degree V_t_/V_0_ of the hydrogel (the ratio of the volume of the formed hydrogel at a certain time point to the initial volume) and time (Figure 5). We found that the hydrogels formed from M1 or M2 contracted rapidly in the first 8 h, when the volume was less than half of the initial volume. After 48 h, the volumes of the samples were about 20% and 30% of the initial volume, respectively. The shrinkage degree was very large. However, the volume of hydrogel formed from M3 still had a volume of more than 98% of the initial volume after 48 h without significant shrinkage. We speculated that the reason why the hydrogel formed by M3 did not shrink significantly may be related to its containing longer OEGMA chain fragments with excellent hydrophilic properties, which could hinder the shrinkage of the hydrogel. In our previous report [29,34], we found that adding a small amount of PEG to the prepared PNIPAM microgel significantly reduced the shrinkage of the resulting hydrogel. Due to the presence of a large number of PEG fragments in OEGMA, we thought that OEGMA may have a similar effect to PEG in reducing hydrogel shrinkage.

We also compared the change of V_t_/V_0_ of the hydrogels formed from M3, M4, and M5 microgels. It was shown that, as the size of P(OEGMA-co-MEO_2_MA) microgel increased, the shrinkage of hydrogel also increased. For instance, after 48 h, the volume of the hydrogel samples formed from M3, M4, and M5 were about 98%, 19% and 17% of the initial volume, respectively. We think the reason was that the larger the particle size of the microgel particles with the same VPTT, the higher the water content. Therefore, when at a certain temperature higher than their VPTT, the shrinkage degree of microgels is greater, resulting in more solvents being extruded from the microgels, which would also cause more volume shrinkage of the macroscopic hydrogels.

These results indicated that, by adjusting the thermosensitivity (VPTT) and particle size of these synthesized P(OEGMA-co-MEO_2_MA) microgels, the properties of the obtained thermosensitive hydrogels can be adjusted, especially volume shrinkage. We also found that the obtained P(OEGMA-co-MEO_2_MA) thermosensitive hydrogels showed a small shrinkage degree only by controlling the synthesis conditions of P(OEGMA-co-MEO_2_MA) microgels; while for the commonly used PNIPAM hydrogels, complex operations, such as physical blending or copolymerization, were required to achieve the same effect. Therefore, the P(OEGMA-co-MEO_2_MA) thermosensitive hydrogel showed better application prospects in the biomedical field. In following work, we will continue to conduct in-depth studies on P(OEGMA-co-MEO_2_MA) microgel- based hydrogels, especially the gelation mechanism, gelation conditions and rheological behavior.

### 2.3. P(OEGMA-co-MEO_2_MA) Thermosensitive Hydrogels for 3D Cell Culture

A hydrogel without shrinkage is more suitable for cell embedding and 3D growth. Therefore, we selected microgel sample M3 as the scaffold material for subsequent 3D cell culture. A type of tumor cell, MCF-7 human breast cancer cell, was chosen as the model cell to explore the feasibility of using this hydrogel to construct a 3D cell model [29,30,33,34,35]. After a certain concentration of microgel dispersion, cells were evenly mixed at room temperature, and then transferred to 48-well culture plates and placed in a cell incubator at 37 °C for culturing. About 1 h later, a stable hydrogel was formed and the cells were encapsulated in situ in it. Part of the DMEM cell culture medium was added to continue this culture process.

**Figure 6:**
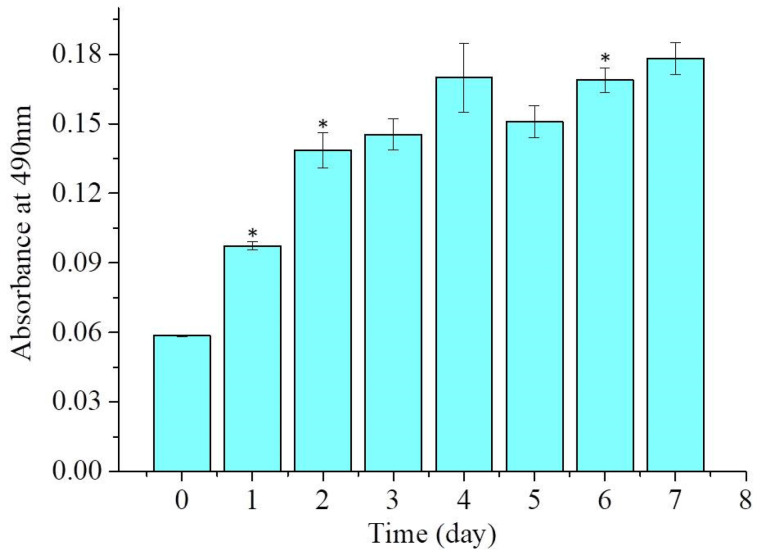
Viability of MCF-7 cells in formed thermosensitive P(OEGMA-co-MEO_2_MA) hydrogel as assessed by MTT assay. The data shown are the mean of three independent experiments. Error bars indicate the standard deviations. An asterisk (*) indicates a significant difference between this group of data and the previous group (*p* < 0.05).

Firstly, we investigated the viability of cells in the P(OEGMA-co-MEO_2_MA) hydrogel scaffold using MTT assay. As shown in Figure 6, the absorbance values measured by the MTT method increased significantly in the first two days of cell culture, which may be related to a rapid increase in the number of living cells in this hydrogel scaffold. This result also indirectly reflected the favorable biocompatibility of this hydrogel scaffold material. The increase of cell viability slowed down after the third day, which may be related to the accumulation of metabolic waste, and the reduction of nutrients and relative living space caused by the increase in the number of cells.

**Figure 7:**
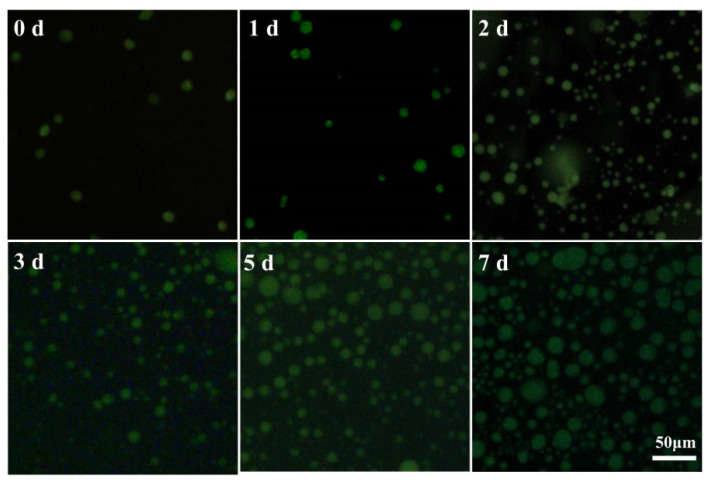
Fluorescence images of MCF-7 cells cultured in the formed thermosensitive P(OEGMA-co-MEO_2_MA) hydrogel. The cells were stained with acridine orange (AO) and ethidium bromide (EB) beforehand.

We further observed the growth and morphology of cells in P(OEGMA-co-MEO_2_MA) thermosensitive hydrogel scaffolds by AO/EB staining, as shown in Figure 7. On the day of cell embedding (day 0), we found that dispersed MCF-7 cells did not adhere to the hydrogel scaffold, but grew uniformly in a single spherical shape in the scaffold. It is well known that cells can only stick to surfaces that are hydrophobic enough, so this cell behavior in the scaffold may be caused by the hydrophilic environment inside the scaffold. After 1 day of culture, some smaller cell clusters could be observed, which may have formed by cell division or cell aggregation. As the culture time was further extended, cells grew faster and the number of cells increased, and most of them showed a green color in the observed field of view, indicating that the cells were still alive and the hydrogel scaffold could maintain the cell growth. When cultured for 7 days, we observed that most of the cells were able to form small multicellular spheres, which may be due to interaction between cells in adjacent cell clusters as the cells continued to spread and grow within the hydrogel scaffold, thus aggregating to form multicellular spheres. Therefore, we can speculate that this type of thermosensitive P(OEGMA-co-MEO_2_MA) hydrogel scaffold can be used to construct tumor multicellular sphere models and may have wide application prospects in drug screening and tumor research. Besides, based on our previous research experience [29], this type of hydrogel scaffold should also be able to be used for 3D culture of many other types of cells, which may extend its range of applications.

## 3. Conclusions

Here, by using MEO_2_MA and OEGMA, having excellent biocompatibility and biodegradability as monomers, we synthesized five types of P(OEGMA-co-MEO_2_MA) thermosensitive microgel samples with different VPTTs or particle sizes by free radical polymerization. Their VPTTs and particle sizes were investigated by means of a nanometer particle size meter and ultraviolet spectrophotometer. With increase of the content of OEGMA, the VPTT of the obtained microgels increased. With decrease of the dosage of SDS, the sizes of the obtained microgels increased. All the types of prepared microgels could aggregate to form 3D hydrogels by thermal induction. All these hydrogels still showed characteristic shrinkage of thermosensitive hydrogels. However, we found that with the increase of the VPTT, or the decrease of the particle size, the less obvious the contraction. Among them, the volume of hydrogel formed from M3 still had a volume of more than 98% of the initial volume after 48 h without significant shrinkage. These hydrogels could be used as a scaffold for 3D culture of MCF-7 model cells and showed excellent biocompatibility. Cells can grow in this scaffold and tend to form multicellular sphere models. This is a simple method for the preparation of thermosensitive hydrogel 3D cell scaffolds, which shows enormous application prospects in the biomedical field, such as tumor research, drug screening, and tissue engineering.

## 4. Materials and Methods

### 4.1. Materials

Oligo(ethylene glycol) methacrylate (OEGMA, Mn = 300), 2-(2-methoxyethoxy) ethyl acrylate (MEO_2_MA), N,N′-methylene diacrylamide (BIS), sodium dodecyl sulfate (SDS), were obtained from Tianjin Heowns Biochemical Technology Co, LTD; potassium persulfate (KPS) was obtained from Tianjin Damao Reagent Factory; Phosphate buffer (PBS), DMEM medium, fetal bovine serum, streptomycin mixture, trypsin, thiazole blue (MTT), acridine orange (AO), ethidium bromide (EB) were obtained from Wuhan Rutgers Biotechnology Co; MCF-7 breast cancer cells were purchased from the Stem Cell Bank of the Chinese Academy of Sciences.

### 4.2. Synthesis and Characterization of P(OEGMA-co-MEO_2_MA) Thermosensitive Microgel

**Scheme 1:**
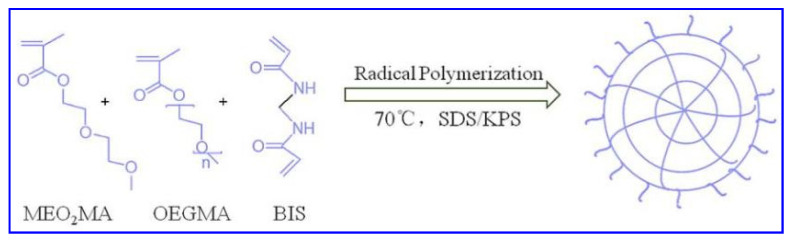
The synthesis of P(OEGMA-co-MEO_2_MA) thermosensitive microgel.

In this manuscript, we first used MEO_2_MA and OEGMA as polymerization monomers, BIS as crosslinker, SDS as surfactant, and KPS as initiator to generate P(OEGMA-co-MEO_2_MA) thermosensitive microgels by free radical polymerization at 70 °C [26] (Scheme 1). The content of BIS was 2%, the mass of KPS were 0.0812 g, respectively. Three types of microgel samples M1, M2 and M3 were synthesized by changing the molar ratios of MEO_2_MA and OEGMA as 95 : 3, 90 : 8, and 85 : 13, respectively. In contrast to the synthesis of M3, another two types of microgel samples, M4 and M5, were synthesized by reducing the SDS dosage, while keeping the other substances constant. The detail feeding amount of each sample is in Table 1.

The typical preparation process for sample M1 was as follows: 2.5002 g MEO_2_MA, 0.1259 g OEGMA, 0.0431 g BIS and 0.0284 g SDS were weighed accurately, dissolved in 100 mL deionized water, and transferred to a three-necked flask. After being passed into N_2_ for 1 h, KPS (dissolved in 5 mL of deionized water) was added to initiate polymerization under magnetic stirring. After 5 h, the reaction was terminated and transferred to a dialysis belt and dialysis for 7 days. Then, the sample was freeze-dried and preserved.

In order to test the thermosensitivities of these samples, the changes of particle size and absorbance (turbidity) with temperature were measured by a nanometer particle size meter and an ultraviolet spectrophotometer, respectively.

### 4.3. Preparation and Characterization of P(OEGMA-co-MEO_2_MA) Microgels-Based Thermosensitive Hydrogel

The thermosensitive P(OEGMA-co-MEO_2_MA) hydrogels were prepared through the thermal induction method [29].

A certain amount of P(OEGMA-co-MEO_2_MA) microgel samples were weighed and dissolved in phosphate buffered saline (PBS) to obtain a final solution with mass fraction of 3.0 wt%. Then, they were placed in a physiological temperature environment, which was higher than their VPTTs. The formation process of thermosensitive hydrogel from microgels and its stability and shrinkage were observed by the inverted method and recorded by camera. The gelation time was defined as the time point at which the inverted hydrogel no longer flowed visually.

To quantitatively analyze the shrinkage degree of the obtained hydrogels, we calculated the ratio of the volume of the obtained hydrogel at certain time point (V_t_) to the initial volume (V_0_) by measuring with a ruler. The thermosensitive hydrogel sample with minimum shrinkage was screened out for the next cell experiments.

### 4.4. P(OEGMA-co-MEO_2_MA) Thermosensitive Hydrogels for 3D Cell Culture

#### 4.4.1. Cell Culture

The model cells used in this experiment were human breast cancer cells (MCF-7), which were cultured in a cell culture incubator using DMEM medium. The main components of the culture medium were 90% DMEM high glucose medium, 10% fetal bovine serum, and 100 unit/mL penicillin/streptomycin double antibodies. The culture temperature was 37 °C and the carbon dioxide concentration was 5%.

#### 4.4.2. Cells Embedded and Cultured in Hydrogel Scaffold

**Scheme 2:**
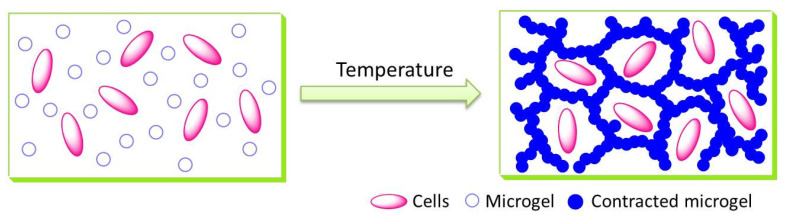
The preparation of the P(OEGMA-co-MEO_2_MA) thermosensitive hydrogel scaffold.

A certain concentration of P(OEGMA-co-MEO_2_MA) microgel solution was prepared and mixed well with an equal volume of the uniform cell suspension to form a mixed solution, in which the cell concentration was about 1 × 10^5^ cells/mL and the microgel concentration was 3.0 wt%. To a 48-well culture plate, 0.5 mL of the above cell/gel mixture was added and placed in a cell culture incubator. With increasing temperature, the solution gelled gradually. After the morphology of the gel in the culture plate was fixed, 0.5 mL of the culture solution was added to each well and recorded as day 0. The culture solution was changed regularly to ensure a nutritious environment for cell growth. The whole process is shown in Scheme 2.

#### 4.4.3. Cell Viability (MTT Assay)

MTT assay was used to detect the viability of cells in the scaffolds. From day 0 of cell culture, three wells were selected each day and 100.0 μL of MTT solution was added to the wells. After continuing to incubate for 4 h, the gel pieces were removed, and 10.0 mL of DMSO was added. Then, the mixture was shaken for 10 min in the dark to dissolve the formed formazan. The absorbance of the solution at 490 nm was measured using a UV-Vis spectrophotometer, and the MTT curve of cells was plotted.

#### 4.4.4. Cell Activity and Morphology (AO/EB Staining)

From day 0 of cell culture, three wells were selected out after culturing for certain days, and one drop of AO/EB double staining solution was added to the selected wells. After further incubation for 1h, the cells were removed, and the morphology and growth state of the cells in the scaffold were observed by an inverted fluorescence microscope.

## Figures and Tables

**Table 1:** The feeding amount of each sample during the preparation process.

Sample	n (MEO_2_MA): n (OEGMA)	m/ g				
		MEO_2_MA	OEGMA	SDS	BIS	KPS
M1	95% : 3%	2.5002	0.1259	0.0284	0.0431	0.0812
M2	90% : 8%	2.3664	0.3368	0.0284	0.0431	0.0812
M3	85% : 13%	2.2371	0.5462	0.0284	0.0431	0.0812
M4	85% : 13%	2.2371	0.5462	0.0284	0.0431	0.0812
M5	85% : 13%	2.2371	0.5462	0.0284	0.0431	0.0812

## Data Availability

Not applicable.
